# Gentamicin Induced Microbiome Adaptations Associate With Increased BCAA Levels and Enhance Severity of Influenza Infection

**DOI:** 10.3389/fimmu.2020.608895

**Published:** 2021-02-23

**Authors:** Yakun Sun, Zhili He, Jiajia Li, Saisai Gong, Shunzong Yuan, Tao Li, Nianzhi Ning, Li Xing, Liangyan Zhang, Fanghong Chen, Zhan Li, Jianxin Wang, Deyan Luo, Hui Wang

**Affiliations:** ^1^ Anhui Medical University, Hefei, China; ^2^ State Key Laboratory of Pathogen and Biosecurity, Beijing Institute of Microbiology and Epidemiology, Beijing, China

**Keywords:** branched-chain amino acids, gut microbiota, myeloid-derived suppressor cell, influenza, T cells

## Abstract

Involvement of gut microbiota in pulmonary disease by the gut-lung axis has been widely observed. However, the cross-talk messengers between respiratory mucosal immunity and gut microbiota are largely unknown. Using selective pharmacologic destruction of gut microenvironment mouse models, we found gut microbiota displayed significantly lower alpha diversity and relative abundance of bacteria in Gentamicin treated mice. Metagenomic studies revealed functional differences in gut bacteria in altering metabolic profiles in mice blood. Branched-chain amino acids (BCAAs) are the essential factors linked between gut and lung. During this process, selective destruction of gut microbiota by Gentamicin induced high levels of BCAAs, and the high levels of BCAAs impacted the lung immunity against influenza virus. *In vivo*, Gentamicin-treated mice or mice fed with high BCAAs diets displayed reduced survival. At the sites of infection, the number of CD11b^+^Ly6G^+^ cells decreased, and CD8^+^ T cells increased accompanied by exuberant expression of pro-inflammatory cytokines could result in tissue damage. CD11b^+^Ly6G^+^ cells transplantation conferred remarkable protection from influenza virus infections. *In vitro*, BCAAs promoted bone marrow-derived cells differentiation to dendritic cells. Taken together, these findings demonstrate that Gentamicin induced disruption of the gut microbiota leads to increased BCAA levels that suppress CD11b^+^Ly6c^+^ cell development in association with overactive CD8^+^ T responses which may contribute to enhanced severity of the viral infection.

## Introduction

**Graphical Abstract:**
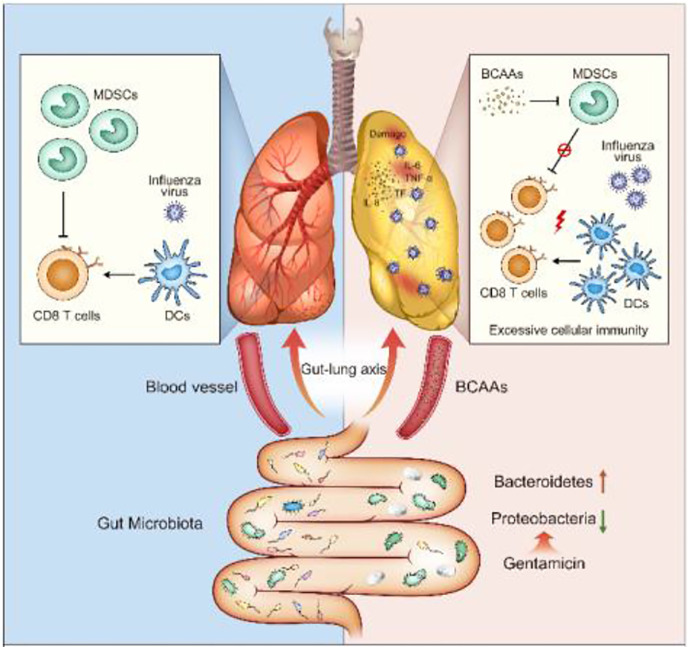


The human intestinal tract harbors trillions of microorganisms referred to as the gut microbiota, which fulfils many essential functions, with disruption of the structure of this community leading to dysbiosis (1–3). Many extrinsic factors, especially broad-spectrum antibiotics became a much-debated topic only recently, can easily alter the microbiota by reducing diversity and shifting community composition (4–7). Several mechanisms by which gut microorganisms can modulate the development of metabolic diseases have been reported (8, 9). Interaction between the host *via* metabolic capacities of the gut microbiota with lung immunity is particular interest for infections (2).

Recently, people have reached a greater understanding of gut-lung axis. This axis from the simple fact that gut microbiota can influence lung function and pathogen clearance mediated by metabolites, microbiota produces, or *via* immune cells and immune factors (10). Respiratory tract infectious diseases, such as influenza and pneumonia, result in the death of 2–3 million people annually worldwide. Understanding the mechanisms that mediate cross-talk between the gut microbiota and lung defenses and how this interaction facilitates optimal lung health is of growing interest. Although, mechanistically, this phenomenon remains poorly defined, the existence of the gut-lung axis and its implications in both health and disease could be profoundly important for lung infection.

Influenza virus is the major source of severe viral respiratory infections, and secondary bacterial infections often follow with influenza infection can cause severe morbidity and mortality involving three to five million people each year (5, 11). Thus, antibiotics were usually used during serious virus infections by clinicians. Recent studies highlight the importance of gut microbiota in shaping lung mucosal immunity (6, 12). However, it remains unclear whether all antibiotics cannot be used during serious virus invasion followed secondary bacterial infections, and whether there is a role of gut microbiota in shaping the lung mucosal immune responses by altering metabolic profiles.

Here, we selected several single antibiotics to set up the conditional alteration of gut microbiota animal models instead of the complete depletion of intestinal flora animal models with giving antibiotics cocktail. The studies revealed that selective disruption of gut microbiota diversity only by Gentamicin increased the susceptibility of lung immunity to influenza virus. Gentamicin is used to kill gram-negative bacteria to induce selective pharmacologic destruction of gut microenvironment and altered metabolism. The Branched-chain amino acids (BCAAs) are found in this list, including leucine, valine, and isoleucine. Cell culture studies show that BCAAs are absolutely essential for several immune cells to synthesize protein and proliferation (13). However, many detailed aspects of BCAAs and its effects on immune function have not been studied. In our study, Gentamicin increased branched-chain amino acids (BCAAs) levels to alter mucosal immunity against influenza infection. This impairment displayed by the mouse models strongly suggests that BCAAs are important cross-talk messengers of gut-lung axis to modulate homeostasis of innate immune response defense against pulmonary infection.

## Materials and Methods

### Mice

All animal studies were conducted in accordance with Beijing Institute of Microbiology and Epidemiology Animal Care and Use Committee guidelines. BALB/c wild type mice (5-week-old, weighting 14–16 g) were obtained from our institute Laboratory Animal Center, Beijing, China. All experimental mice were bred in a specific pathogen-free facility at our institute. Experimental mice were matched for age and sex and cared for according to guide lines of institute. Mice were monitored and weighted at least once daily after initiating infection. Recumbent mice, and mice that lost more than 30% weight, were considered moribund and euthanized.

### Virus Infection and Mouse Treatment

Influenza A virus (IAV) A/Puerto Rico/8/1934 (PR8) was propagated in 10-day-old specific pathogen-free chicken embryos. Where indicated, mice were treated pharmacologically by supplementing drinking water with 160 ^u^/ml Gentamicin, or 5 mg/ml Streptomycin, or 0.05 mg/ml Vancomycin, or 0.5 mg/ml Tinidazole (Sigma-Aldrich, USA) beginning 3 days prior to infection with replenishment every 48 h. Mice were supplied with high-BCAAs diets (5 mg/day, the ration of three amino acids is 1:1:1) and normal diets daily beginning 3 days prior to infection. Infections were performed by applying 10^3.5^TCID_50_ influenza PR8 intranasally. All virus infections were performed in BLS-2 laboratory and followed the specific procedures.

### 16S rDNA Amplicon Sequencing

Total genome DNA was extracted using CTAB/SDS method from colonic contents. DNA concentration and purity were monitored on 1% agarose gels. 16S rDNA/ITS genes of distinct regions (16SV4/16SV3/16SV3-V4/16SV4-V5, ITS1/ITS2, Arc V4) were amplified used specific primer (e.g. 16S V4: 515F-806R, et al.) with the barcode. All PCR reactions were carried out with Phusion^®^ High-Fidelity PCR Master Mix (New England Biolabs). Samples with bright main strip between 400 and 450 bp were chosen for further experiments. Then, mixture PCR products were purified with Qiagen Gel Extraction Kit (Qiagen, Germany). Sequencing libraries were generated using TruSeq^®^ DNA PCR-Free Sample Preparation Kit (Illumina, USA) following manufacturer’s recommendations and index codes were added. The library quality was assessed on the Qubit@ 2.0 Fluorometer (Thermo Scientific) and Agilent Bioanalyzer 2100 system. At last, the library was sequenced on an IlluminaHiSeq2500 platform and 250 bp paired-end reads were generated.

### Nuclear Magnetic Resonance Metabonomics Analysis

NMR metabonomics analysis was done by Wuhan Anachro. In brief, serum of mice treated with antibiotic and controls were vortexed, aqueous layer was transferred to 0.5 ml 3KDa ultrafiltration filter (Millipore, USA). Fifty microliters H2O and 50 μl DSS standard solution (Anachro, Canada) was added. Samples were mixed well before transfer to 5mm NMR tube (Norell, USA). Spectra were collected using a Bruker AV III 600 MHz spectrometer equipped with an inverse cryoprobe. The first increment of a 2D-1H, 1H-NOESY pulse sequence was utilized for the acquisition of 1H-NMR data and for suppressing the solvent signal. Experiments used a 100 ms mixing time along with a 990 ms pre-saturation (~80 Hz gammaB1). Spectra were collected at 25°C, with a total of 64 scans over a period of 7 min. The collected Free Induction Decay (FID) signal was automatically zero filled and Fourier transform in Processing module in Chenomx NMR Suite 8.1. (Chenomx Inc., Edmonton, Canada). The data were then carefully phased and baseline corrected by experienced technician in Chenomx Processor. All the spectra were referenced to the internal standard, DSS and analyzed by experienced analysts against Chenomx Compound Library. All metabolites’ concentration information was exported to excel and normalized by weight across all parallel samples before used in the later on multivariable analysis. PCA and PLS-DA were performed using the pcaMethods bioconductor package (14) and pls package respectively. Plots were made using ggplot2 package (15).

### Measurements of Virus Copy and Immune Parameters

Total RNA was obtained from lung tissue with TRIzol reagent (Invitrogen). The cDNA was generated by reverse transcription with commercial PrimeScript RT Master Mix (Takara). Tissue levels of mRNA encoding TNF-α, IL-6, IL-1β, IFN-γ, IL-8, and TF were measured by real-time PCR (LightCycle 480), normalized to levels of mRNA encoding β-actin, and expressed as fold change relative to levels in uninfected wild-type mice. The influenza viral burden in whole lung tissue was determined also by real-time PCR measuring influenza virus membrane protein 1 (M1) copy number. The primer pairs used for real-time PCR were designed using Primer.

**Table:** 

	Forward	Reverse
TNF-α	CATCTTCTCAAAATTCGAGTGACAA	TGGGAGTAGACAAGGTACAACCC
IL-6	GAACAACGATGATGCACTTG	TGAAGGACTCTGGCTTTGTC
IL-1β	GAACAACGATGATGCACTTG	TTCTTTGGGTATTGCTTGGGA
IFN-γ	CATTGAAAGCCTAGAAAGTCTGAATAAC	TGGCTCTGCAGGATTTTCATG
IL-8	TGGCAGCCTTCCTGATTT	AGGTTTGGAGTATGTCTTTATGC
TF	CAATGAATTCTCGATTGATGTGG	GGAGGATGATAAAGATGGTGGC
M1	AAGACCAATCCTGTCACCTCTG	CAAAACGTCTACGCTGCAGTCC
β-actin	GGAGGGGGTTGAGGTGTT	GTGTGCACTTTTATTGGTCTCAAG

Total IgGs titers of serum specific to PR8 virus were measured by enzyme-linked immunosorbent assay (ELISA). The PR8 virus were freeze thawed repeatedly, then coated in 96-well plates overnight at 4°C. Serum samples were serially diluted in 2-fold dilutions from 1:10 to 1:20480. The endpoint dilution titer was calculated as the serum dilution resulting in an absorbance reading of 0.2 units above background. Goat anti-mouse IgG-HRP (Sigma, 1:5000) was used as the detection antibodies. The reactions were developed with TMB(3,3’,5,5’-Tetramethylbenzidine) and stopped with 2 M H_2_SO_4_. The absorbance at 450 nm was detected.

### Flow Cytometry

Preparation of lung single cells was described by Dr Smiley (16). In brief, cells isolated from lung tissues were ground with glass rod and digested with collagenase, and then single cells were incubated with Fc Block (clone 2.4G2) for 15 min at 4°C, washed 3 times. For enumeration of CD45 cells, macrophages, and neutrophils, pulmonary lymphocytes were stained on ice with anti-CD45-EF450 (cloneRM-5), anti-CD11b-APC (clone M1/70), and anti-Ly6G-FITC (clone 1A8), anti-Gr-1-Alexafluor700 (RB6-8C5), anti-CD3-PerCP-eFlour™-710 (clone17A2), anti-CD4-eFlour^®^450 (cloneRM4-5), anti-CD8a-PE-Cy7 (clone53-6.7) (eBioscience, USA). Data were gated for forward scatter/side scatter and collected on an FACSCanto II (BD Biosciences) and analyzed using FlowJo software (Tree Star).

### Spleen CD11b^+^Ly6G^+^Cells Isolation and Adoptive Transfer

The method for CD11b^+^Ly6G^+^cells purification is supplied by Dr Tsukasa Seya’s team (17). CD11b^+^Ly6G^+^ cells were isolated from a single cell suspension from the spleen of wild-type mice by using a biotin-conjugated anti-Ly6G monoclonal Ab (1A8) (Biolegend, San Diego, CA, USA) and Streptavidin Microbeads (MiltenyiBiotec, Bergisch Gladbach, Germany) according to the manufacturer’s instructions. In these purification steps, two rounds of positive selection were performed to increase purity. We routinely prepared Ly6G^+^cells at >95% purity and almost 100% of Ly6G^+^ cells expressed CD11b. Isolated CD11b^+^Ly6G^+^cells were resuspended in PBS, and then adoptive transferred intravenously into recipient mice (5 × 10^5^ viable cells/mouse), which were challenged with 10^3.5^TCID_50_ (mice treated with Gentamicin 3 days) influenza 3 days before CD11b^+^Ly6G^+^ cells transfer.

### CD11b^+^Ly6G^+^ Cells Induction From Bone Marrow Stromal Cells

The method of bone marrow stromal cells preparation is described by Dr Dina Sabry (18). In brief, Tibia and fibula bone marrow was flushed out with phosphate-buffered saline (PBS) containing 2 mM EDTA for isolation and culture. Then, the sample was layered carefully on Ficoll-Paque (Gibco-Invitrogen, Grand Island, NY), and keep the mononuclear cell layer for the next study. Cells were stimulated *in vitro* in bulk culture with granulocyte-macrophage colony stimulating factor (GM-CSF) and interleukin-4 (IL-4). In brief, thirteen days after initiation of culture, the culture was replenished with an equal volume of medium containing 50 ng/ml recombinant human GM-CSF every other day. After 5 days of culture, the cultures were replenished with an equal volume of medium containing 50 ng/ml recombinant human IL-4 and/or different concentration of BCAAs (5 and 1 µg/µl, the ration of three amino acids is 1:1:1) (Qianrun Inc. China) every other day. After 3 days of culture, cells were harvested and evaluated by Western-blot and Flow cytometer.

### Western-Blot

Bone marrow stromal cells were cultured with GM-CSF/IL-4 and different concentration of BCAAs/rapamycin (Sigma-Aldrich, USA). Cells were harvested and analyzed by 8% SDS-PAGE. Samples separated in the gel were electro transferred to a PVDF membrane (GE). This step was followed by the antigen-antibody reactions. Rabbit anti-mTOR hyper-immune sera was used as the detecting antibody, and horseradish peroxidase-conjugated goat anti-rabbit immunoglobulin G (IgG) served as the secondary antibody (Cell Signaling Technology Inc). Following the addition of the substrate diaminobenzidine, the specific protein bands were revealed.

### Histology

Lung tissues were fixed in 10% neutral buffered formalin, embedded in paraffin, sectioned, and stained with hematoxylin and eosin. The pathological foci in each section were evaluated. (i.e. areas with large numbers of inflammatory cell infiltration accompanied by evidence of edema of submucosa). Representative photomicrographs depict × 100 magnification.

### Statistical Analysis

Statistical analyses were performed using the program Prism 5.0 (GraphPad Software, Inc., La Jolla, California, USA). Values are expressed as mean ± SD. Data were analyzed by unpaired Student’s t-test (normal distribution) or one-way ANOVA followed by Dunnett’s multiple comparison tests. Survival data were analyzed by log rank tests. Statistical analysis of microbiota data was performed in Rhea (19). EzTaxon (20) was used for the identification of OTUs showing significant differences (p<0.05) in relative abundances between feeding groups. MetPA. Pathway analysis was launched by MetaboAnalyst 3.0 using identified differential metabolites (variables with VIP of >1 or P< 0.05 in one-way ANOVA). p<0.05 was considered to be statistically significant.

## Results

### Conditional Alteration of Gut Microbiota Increased Susceptibility to Influenza Virus Infection in Lung

**Figure 1:**
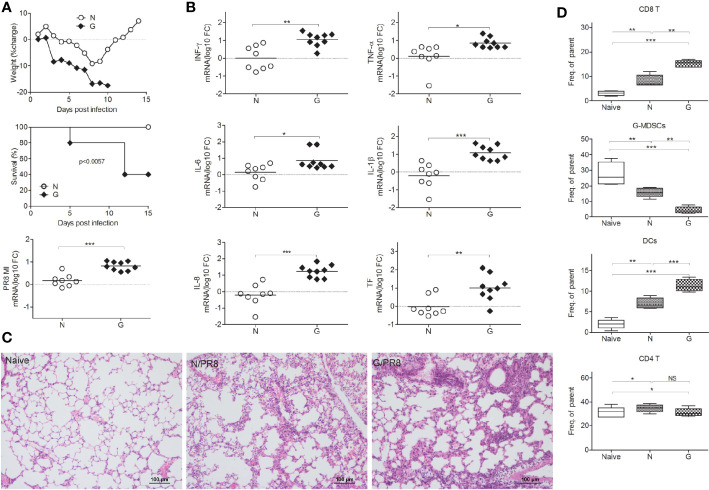
Gut microbiota influences susceptibility to influenza virus infection in lung. Gentamicin treated mice and control mice were inoculated with a sublethal dose (10^3.5^TCID_50_) of influenza PR8, and monitored survival and weighted mice every day. On day 8 after the inoculations, mice were euthanized, **(A)** percent body weight change, survival, lung virus titer and **(B)** lung levels of mRNA encoding IFN-γ, TNF-α, IL-6, IL-1β, IL-8, and TF were measured (n=8-10 per group). Gut microbiota helped to reduce pathology during infection **(C)**, naïve mice (Naïve), mice infected with virus (N/PR8), mice treated with Gentamicin and then infected with virus (G/PR8). **(D)** CD8^+^ T cells, CD11b^+^Ly6G^+^ cells (G-MDSCs), CD4^+^ T cells, and DCs were evaluated by Flow cytometry. *<0.05, **p<0.01, ***p<0.001. N, none, G, Gentamicin.

To investigate the role of gut microbiota diversity altered by different antibiotics during influenza virus infection, mice were treated with antibiotics (Streptomycin, Vancomycin, Tinidazole, and Gentamicin, all antibiotics cannot be absorbed easily by intestine) separately, and then intranasal challenged with sublethal dose (10^3.5^TCID_50_) Influenza A virus (A/Puerto Rico/8/1934, PR8). This single antibiotic treated animal models are totally different from mice giving antibiotics cocktail for depletion of intestinal flora completely. We found only Gentamicin can cause mice dead (**Supplementary 1-1**, **Figure 1A**). Control mice readily survived sublethal dose intranasal challenge with 10^3.5^TCID_50_ influenza virus PR8 (**Figure 1A**). Parallel evaluations of body weight changes over the course of infection suggested that the control mice experienced less severe disease than did mice treated with Gentamicin (**Figure 1A**). This susceptibility correlated with increased virus titers in the lung. Meanwhile, we also challenged Gentamicin treated mice with sublethal dose of H5N1 influenza virus, and then measured susceptibility to this subtype influenza virus. We observed that Gentamicin treated mice also succumbed to sublethal dose of H5N1 influenza challenge (data not shown). Notably, susceptibility to influenza virus infection did correlate with gut microbiota diversity. In our study, significant differences were observed in the diversity and composition of gut microbiota in antibiotic treated mice (**Supplementary 2-1**). Analysis of mice gut microbiota showed significantly lower alpha diversity and relative abundance (p<0.01) of bacteria in antibiotic treated mice(data not shown). Mice treated with Gentamicin shifted intestinal community by increasing *Bacteroidetes* and decreasing *Proteobacteria* (**Supplementary 2-1**). To further investigate the impact of gut microbiota mediated protection from influenza virus infection, we evaluated pro-inflammatory responses and tissue damage in the lung. We measured inflammation markers and pathology in the lung of mice treated with Gentamicin and control mice at day 8 after inoculation of sublethal dose of influenza virus PR8. Consistent with our previous discoveries of virus titers (**Figure 1B**), Similar trends were observed for markers of inflammation, including levels of mRNA encoding IFN-γ, TNF-α, IL-6, IL-1β, IL-8, and TF. Following levels of inflammatory markers detection, we also assayed the pathological score after the virus infection. In Gentamicin treated mice, the cellular infiltrates tended to be medium to large and were frequently associated with large areas of pulmonary edema, whereas the foci in the control mice tended to be small to medium, with little evidence of edema (**Figure 1C**). Pathological scoring revealed significant differences in the number and quality of pulmonary foci showing high degrees of edema.

CD8^+^ T cells can facilitate the major protection against influenza strains, thus, cellular immunity to influenza might be predicted to exacerbate susceptibility to respiratory infection disease (21–23). However, depletion of CD8^+^ T cells diminished the protection against influenza virus infection (24). Thus, maintaining the appropriate levels of T cell responses is very important during virus infection. To test the role of immune cells in defense against virus-induced respiratory susceptibility, we administered Gentamicin to naive mice prior to infection with PR8 influenza, and then tested immune cell responses. We observed that the number of pulmonary CD8^+^T cells (**Figure 1D**, **Supplementary 1-2A**) increased, CD11b^+^Ly6G^+^ cells (**Figure 1D**, **Supplementary 1-2B**) decreased and dendritic cells (DCs) (**Figure 1D**, **Supplementary 1-2C**) increased significantly in Gentamicin-treated mice. CD11b^+^Ly6G^+^ cells are identified as granulocytic myeloid derived suppressor cells (G-MDSCs). Just like in tumor mouse models, we believe that the abundance of CD11b^+^Ly6G^+^ cell is the important role of regulation CD8^+^ T cell responses during acute virus infections. This subtype myeloid-derived suppressor cells (MDSCs) are responsible for the suppression of T cell-mediated immunity that induced tissue damage.

### Gentamicin Treatment Altered BCAAs Levels

**Figure 2:**
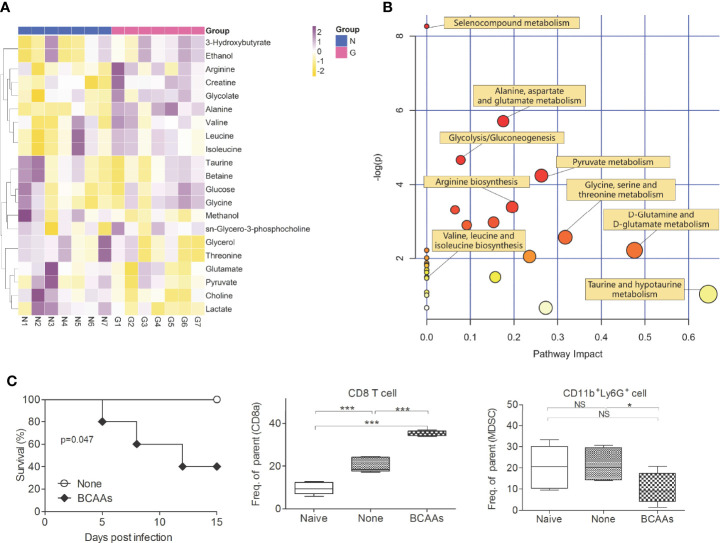
Gentamicin-induced alteration of metabolomics profiles. Serum (n=7) were isolated form mice treated with Gentamicin (G) and none (N), and then analyzed the metabolomics profile. Hierarchical clustering of identified differential metabolites (variables with VIP of >1 or P< 0.05 in one-way ANOVA) **(A)** were shown by heatmap. Each row shows relative ion intensity for a specific metabolite after mean centering and unit variance scaling of the data. Each column shows the serum metabolic profiles of the two groups. **(B)** MetPA. Pathway analysis was launched by MetaboAnalyst 3.0 using identified differential metabolites (variables with VIP of >1 or P< 0.05 in one-way ANOVA). The color and size of each circle are based on its P-value and pathway impact value, respectively. **(C)** Survival, CD8^+^T cells and CD11b^+^Ly6G^+^ cells (G-MDSCs) were shown in mice supplied with high-BCAAs diets, and then intranasally challenge with sublethal dose influenza virus. *<0.05, **p<0.01, ***p<0.001. NS, no significant.

To investigate the importance of diversity of gut microbiota and metabolic profile during virus infections, the High-throughput 16S rDNA gene amplicon analysis and nuclear magnetic resonance (NMR) metabonomics analysis were used. Significant differences were observed in the diversity and composition of gut microbiota in Gentamicin treated mice. Analysis of mice gut microbiota showed significantly lower alpha diversity and relative abundance (p<0.01) of bacteria in Gentamicin treated mice (**Supplementary 2-1**), such as *Bacteroidetes*, *Proteobacteria* and so on. Metagenomic studies revealed functional differences in gut bacteria in altering metabolic profiles in mice blood (**Supplementary 2-1**). **Supplementary 2-2** indicates that 21 compounds were identified in Gentamicin treated samples (n=7), including 7 amino acids, glucose, choline, lactate and so on. After filtering the metabolites [variable importance in projection (VIP) value of >1 or P< 0.05 in intergroup comparisons by one-way analysis of variance (ANOVA)], hierarchical clustering showed different metabolic signatures between groups (**Figure 2A**). A metabolic pathway analysis (MetPA) of altered metabolites was performed by using the MetaboAnalyst 3.0 online tool (25), revealing the change of 16 compounds metabolism pathway to be pronounced (**Figure 2B**). The Branched-chain amino acids (BCAAs) are found in this list, including leucine, valine, and isoleucine. To further investigate which compound plays the key role defense against influenza infection, we assayed some important compounds *in vivo.* We found the function of BCAAs during influenza virus infection. BCAAs are known to play positive roles during host immunity to bacterial infection (13). Indeed, in our studies, mice administered with Gentamicin markedly increased levels of BCAAs in blood, whereas these mice succumbed to sublethal dose influenza virus challenge (**Figure 1B**). So we believe BCAAs can “modulate” immune homeostasis in different models. To assess the role of BCAAs played during virus infection, mice were supplied with high-BCAAs diets and normal diets, and then intranasally challenge with sublethal dose influenza virus. Consistent with our observation we did in Gentamicin treated animal model, we also observed that mice succumbed to virus challenge when supplied with high-BCAAs diets (**Figure 2C**), and the number of pulmonary CD8^+^ T cells increased and CD11b^+^Ly6G^+^ cells (G-MDSCs) decreased significantly. Thus, gut microbiota mediated high level of BCAAs may have influenced CD11b^+^Ly6G^+^ cells’ function, and the disruption of immune balance between CD8^+^ T cells and CD11b^+^Ly6G^+^ cell exacerbated susceptibility to respiratory infection disease.

### BCAAs Levels Mediated Differentiation of Bone Marrow-Derived Cells (BMDCs) *via* mTOR Contributes to CD11b^+^Ly6G^+^ Cells Exhausting

**Figure 3:**
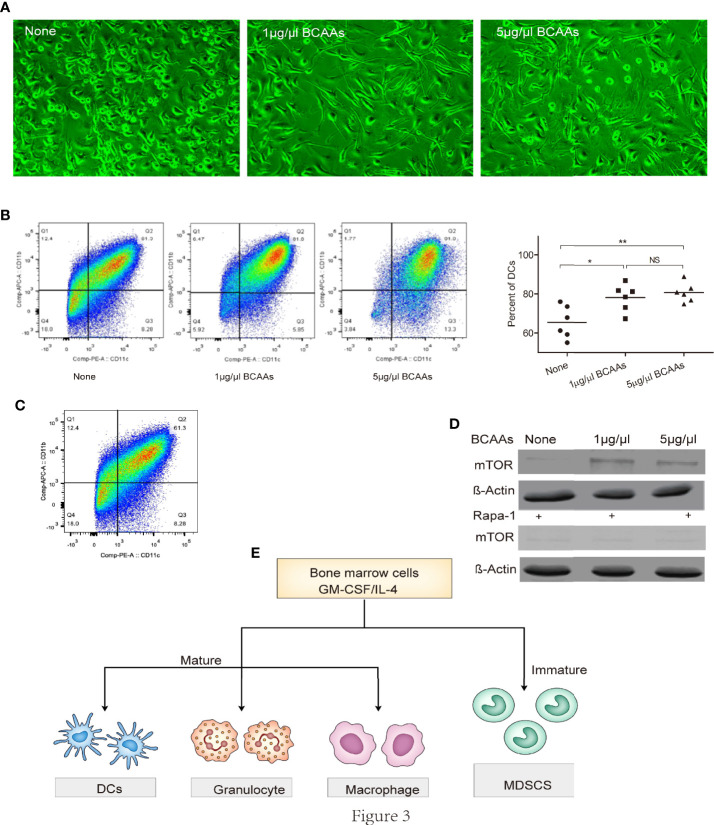
BCAAs are essential to promote BMDCs differentiation into DCs. Bone marrow-derived cells were isolated and examined the differentiation or proliferation under different concentration of BCAAs *in vitro*. **(A)** Cell morphology under microscope, **(B)** Percent of DCs, **(C)** CD11b^+^Ly6G^+^ cells induction from BMDCs, **(D)** Western-blot of mTOR, **(E)** Mechanism of BMDCs differentiation. N, none, G, Gentamicin. All assays were repeated at least three times. *<0.05, **p<0.01, ***p<0.001. NS, no significant.

To investigate this possibility, we isolated BMDCs and examined the proliferation under different concentration of BCAAs *in vitro*. We found that cellular morphology was different after BMDCs were treated with BCAAs (**Figure 3A**). The flow cytometry was used to identify the percent of DCs, macrophages and granulocytes under different concentration of BCAAs. With the same induction method, we found only DCs increased (**Figures 3B, C**) when BMDCs were treated with BCAAs. These results were also found *in vivo* when mice treated with Gentamicin (**Figure 1D**). All findings suggest that BCAAs were important for functional MDSCs differentiation. BCAAs involve activation of mammalian target of rapamycin (mTOR) pathway, which mediates multiple cellular functions, including controlling the maintenance and function of T-reg cell in the periphery (26, 27). Cell culture studies show that BCAAs are absolutely essential for several immune cells to synthesize protein and proliferation. To investigate the mechanism involved in BCAAs induced functional MDSCs differentiation, we evaluated mTOR expression during CD11b^+^Ly6G^+^ cells proliferation or differentiation. We found that the mTOR expression in BMDCs were promoted by BCAAs and inhibited by rapamycin (**Figure 3D**). We believe that BCAAs impacted mTOR expression to induce BMDCs to differentiate into mature DCs in pulmonary microenvironment. **Figure 3E** depicts the proliferation and differentiation of BMDCs. Immature myeloid cells are part of the normal process of myelopoiesis, which takes place in the bone marrow and includes monocytic MDSCs (M-MDSCs) and G-MDSCs. Normally, the ability to differentiate into mature DCs and macrophages has been shown to be restricted to M-MDSCs. Our results suggested that BCAAs levels with pulmonary microenvironment induced BMDCs to differentiate into DCs maybe through M-MDSCs, which will result in G-MDSCs reduction.

### Adoptive Transfer With Ly6G^+^CD11b^+^ Cells That Confer Protection Against Pulmonary Influenza Virus Infection

**Figure 4:**
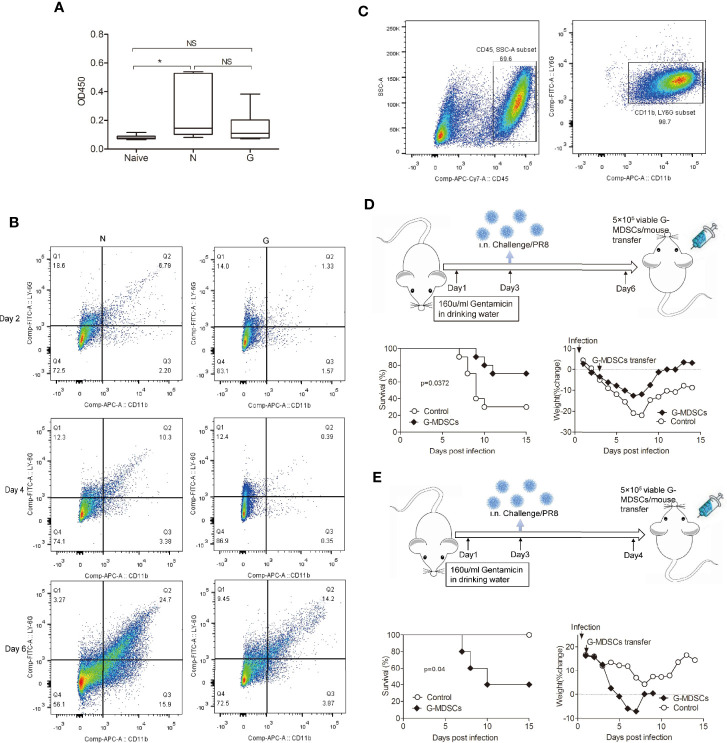
Inhibition of exuberant T cell responses and inflammation by purified CD11b^+^Ly6G^+^ cells. **(A)** Wild-type mice were injected with anti-Ly6G-specific mAb and anti-CD11b-specific mAb, most mice died with influenza virus challenge. **(B)**Time course of CD11b^+^Ly6G^+^ cell numbers in Gentamicin treated mice after challenged with influenza. **(C)** CD11b^+^Ly6G^+^ cells were purified from spleen single cells. **(D)** Mice treated with Gentamicin were transferred with CD11b^+^Ly6G^+^ cells on day 3 after sublethal dose influenza virus infection, survival and percent body weight changes. **(E)** Same mice were transferred with CD11b^+^Ly6G^+^ cells on day 1, survival and percent body weight change. N, none/PR8, G, Gentamicin/PR8. All assays were repeated 2 times *<0.05, **p<0.01, ***p<0.001. N, none; G, Gentamicin; NS, no significant.

We used virus infection model for our research, one very important immune readout is viral specific IgGs responses. To assess whether viral specific IgGs titers also played an important role in mice treated with Gentamicin, serum samples were collected on day 8 after infection. We did not find any differences in total IgG titers between mice treated with/without Gentamicin (**Figure 4A**).

To investigate whether CD11b^+^Ly6G^+^ cells could suffice to protect against acute influenza virus infection, infected mice were transferred with CD11b^+^Ly6G^+^ cells. Before the cell transfer, we evaluated the time course of CD11b^+^Ly6G^+^ cells accumulation during influenza infection in Gentamicin-treated mice (**Figure 4B**). We found that the number of CD11b^+^Ly6G^+^ cells kept lower in Gentamicin-treated mice in comparation with normal controls, and bottom on day 3 or on day 4. We believe day 3 or day 4 is the best window period for cell transplantation. So, we purified CD11b^+^Ly6G^+^ cells (**Figure 4C**) according to the method supplied by Dr Tsukasa Seya’s team for the next step. Initially, wild-type mice were treated with Gentamicin and then challenged with sublethal dose of influenza virus intranasally. After 3 days, we employed an adoptive transfer strategy in which CD11b^+^Ly6G^+^ cells were purified from spleen, and then injected 5 × 10^5^ viable cells intravenously into mice. In comparison with mice transferred with microbead only, mice transferred with CD11b^+^Ly6G^+^ cells displayed modest but significant protection (p<0.05) after challenged with sublethal dose of influenza, as evidenced by both a 2-day prolongation in median survival time and an increase in the overall survival rate to 40% (**Figure 4D**). To further investigate whether the protection conferred by adoptive transferred with CD11b^+^Ly6G^+^ could be used for acute virus infection anytime or any condition, we transferred 5 × 10^5^ viable cells intravenously into recipients without Gentamicin treatment. Control mice received microbeads alone. We observed opposite results (**Figure 4E**), which suggested that CD11b^+^Ly6G^+^ cells could suffice to protect against acute virus infection when the homeostasis of immune cells was disrupted.

## Discussion

Antibiotics have been proposed as supplements in re-feeding program for malnourished children. A review of pediatric literature showed that growth promotion by antibiotics (7), when it was observed, was mostly mediated by its anti-infective properties. Despite the widespread use of antibiotics as growth promoters in animal rearing, the available evidence again points to the suppression of infections as the underlying mechanism (4). The potential clinical relevance of the devastating effect of antibiotics on the gut microbiota has become a much-debated topic only recently. Therefore, scientists set antibiotics treated animal models to address the influence of gut microbiota to lung, most of them gave antibiotics cocktail to mice to kill bacteria almost completely (12). We were curious if all antibiotics could induce disruption of the structure of gut microbiota leading to dysbiosis. Here, we treated mice with different kind of antibiotics to address it. All antibiotics can alter the diversity of gut microbiota definitely. However, only Gentamicin treated mice increased susceptibility to influenza virus infection. Gentamicin was used as an oral medicine for diarrhea worldwide more than 10 years ago. Usually, clinicians use it as a very safe antibiotic only because it can’t be absorbed by intestine. But the FDA reported in 2019 that influenza infection was found among people who take Gentamicin, especially for people who are 2–9 years old. In our study, mice treated with Gentamicin shifted intestinal community by increasing *Bacteroidetes* and decreasing *Proteobacteria*. This feature of intestinal commensal flora altered by Gentamicin is very special for influenza infection.

Because we gave mice Gentamicin only 3 days, it was difficult that gut microorganisms could colonize in respiratory tract in this short period (28). We concluded by drawing attention to the interactive messengers between gut and lung which were influenced by altered gut microbiota. In the present study, we demonstrated that BCAAs control the maintenance and function of CD11b^+^Ly6G^+^ myeloid-derived suppressor cells in the lung tissue, and BCAAs may promote mTOR activity during DCs differentiation from BMDCs. Although CD11c^+^/CD11b^+^ DCs express high major histocompatibility class II (MHC-II), these cells still can present endogenous antigens to T cells by MHC-I/MHC-II cross-presentation. Ikeda et al. find that BCAAs are essentially required to maintain expansion and suppressive capacity of Treg cell *via* mTOR, which regulate excess immune responses (27). In tumor models, CD11b^+^Ly6G^+^ myeloid-derived suppressor cells also be considered as a regulatory cell subtype to suppress T cell responses (29, 30). Thus, it looks like that BCAAs are important to modulate immune homeostasis. Usually, people take BCAAs to make their muscle strong. However, during epidemics of seasonal influenza, people should take BCAAs discreetly.

Inflammatory responses are initiated in response to pathogen infection, contributing to protective host immunity. However, failure to control exuberant expression of pro-inflammatory cytokines can result in tissue damage (31, 32). In our study, we detected high levels of pro-inflammatory cytokines expression and inflammatory foci with various degrees of lung damage in virus infected mice treated with Gentamicin. Elevated proliferation of the high levels of CD8^+^ T cells and low levels of CD11b^+^Ly6G^+^ myeloid-derived suppressor cells have been associated with enhanced inflammation (33). MDSCs are an immunosuppressive subset of cells that arise from myeloid progenitors (34, 35), and expressing both CD11b^+^ and Ly6G^+^ surface markers cell subtype represents a population of tumor-supporting myeloid cells (36). CD11b^+^Ly6G^+^ cells were shown to produce reactive oxygen species/reactive nitrogen species including hydrogen peroxide and peroxynitrite (PNT). PNT is responsible for the suppression of T cell-mediated immunity that enable tumor cells to escape tumor antigen-specific CTLs (37, 38). However, recent studies have revealed that functionally polarized CD11b^+^Ly6G^+^ cells that means this cell subtype has both a positive and negative impact on tumor growth (39, 40). Meanwhile, the function of MDSCs during acute infection still keep unknown. Thus, a full understanding of the phenotype and function of each of these cell populations is required in order to understand the mechanisms that clear pathogens, prevent systemic spread, and reduce immune-mediated tissue damage.

Because we have no way to decrease the levels of BCAAs in our animal models, we presume if we can use CD11b^+^Ly6G^+^ cells transplantation which levels were influenced by BCAAs instead. Our observations demonstrated that adoptive transfer with CD11b^+^Ly6G^+^ cells that conferred remarkable protection from influenza virus infections. Under certain conditions, CD11b^+^Ly6G^+^ cells can dampen the inflammatory response through inhibiting T cell responses (41), and CD11b^+^Ly6G^+^ cells will be used clinically as an anti-inflammatory cell subtype, especially during acute virus infection.

Studies over the past a few years have revealed remarkable interplay between microbiota, immunity, and infection. This report extends those connections by demonstrating that gut microflora leading to changing metabolic characterization can critically influence the homeostasis of immune response against acute virus infection.

## Data Availability Statement

The datasets presented in this study can be found in online repositories. The names of the repository/repositories and accession number(s) can be found in the article/**Supplementary Material**.

## Ethics Statement

The animal study was reviewed and approved by Beijing Institute of Microbiology and Epidemiology Animal Care and Use Committee guidelines.

## Author Contributions

All authors discussed the results and implications of the manuscript. HW and DL conceived the study, supervised the project, analyzed data, and wrote the paper. YS, ZH, JL, SG, SY, TL, NN, LX, LZ, FC, ZL, and JW performed experiments and analyzed data. DL advised on statistical evaluations. YS and ZH contributed equally to this work. All authors contributed to the article and approved the submitted version.

## Funding

This work was supported by National Key Basic Research Program (973) (2015CB554202), National Key Research Project (2018ZX10101003-005-001), National Natural Science Foundation of China (31870156), National Key Lab Research program (SKLPBS1811).

## Conflict of Interest

The authors declare that the research was conducted in the absence of any commercial or financial relationships that could be construed as a potential conflict of interest.

## Supplementary Material

Supplementary 1-1 Different antibiotics treatment followed by influenza virus infection. Antibiotics treated mice and control mice were inoculated with a sublethal dose (10^3.5^TCID_50_) of influenza PR8. On day 8 after the inoculations, mice were euthanized, **(A)** Survival, **(B)** Percent body weight change.

Supplementary 1-2 Flow cytometry analysis of lung cells of mice treated with Gentamicin and then infected with influenza. Gentamicin treated mice and control mice were inoculated with a sublethal dose (10^3.5^TCID_50_) of influenza PR8. On day 8 after the inoculations, mice were euthanized, **(A)** CD8^+^ T cells, **(B)** CD11b^+^Ly6G^+^ cells, and **(C)** DCs. N, none/PR8, G, Gentamicin/PR8.

Supplementary 2-1 Gentamicin-induced alteration of colonic microbiota profiles. **(A)** Tags and outs **(B)** Relative abundance in phylum levels. **(C)** Extended error bar plot showing the two bacterial genera with a significant difference (T-test; P< 0.05) in proportions of at least 1% between samples of control group (N) and samples of Gentamicin-treated group (G). One genera(*Bacteroidetes*)is over abundant within the colonic microbiotas collected from mice treated with Gentamicin compared to those collected from control mice, genera abundance (left) and 95% confidence intervals (right), were detected in 7 Getamicin-treated mice. Number of mice: Control, 7; Gentamicin treated group, 7. N, none/PR8, G, Gentamicin/PR8.

Supplementary 2-2 Metabolite assignments and chemical shifts of distinguishable peaks.
