# Supplementary figures and images for: Gentamicin Induced Microbiome Adaptations Associate With Increased BCAA Levels and Enhance Severity of Influenza Infection
Source: Front Immunol. 2021 Feb 23;11:608895. doi: 10.3389/fimmu.2020.608895 (PMC7940682; doi:10.3389/fimmu.2020.608895)

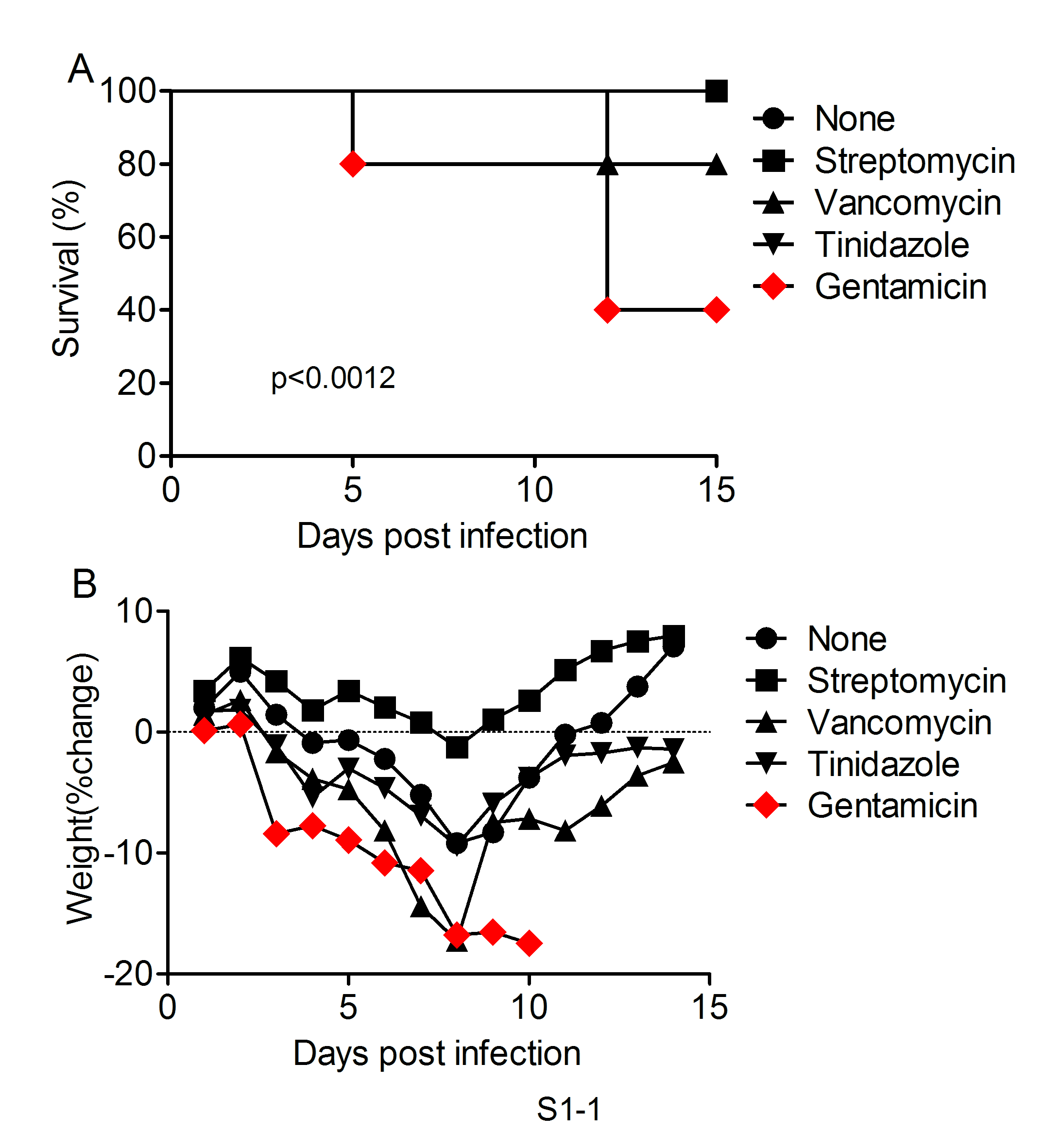

Supplement: Supplementary 1-1 — Different antibiotics treatment followed by influenza virus infection. Antibiotics treated mice and control mice were inoculated with a sublethal dose (103.5TCID50) of influenza PR8. On day 8 after the inoculations, mice were euthanized, (A) Survival, (B) Percent body weight change. [file Image_1.tif]

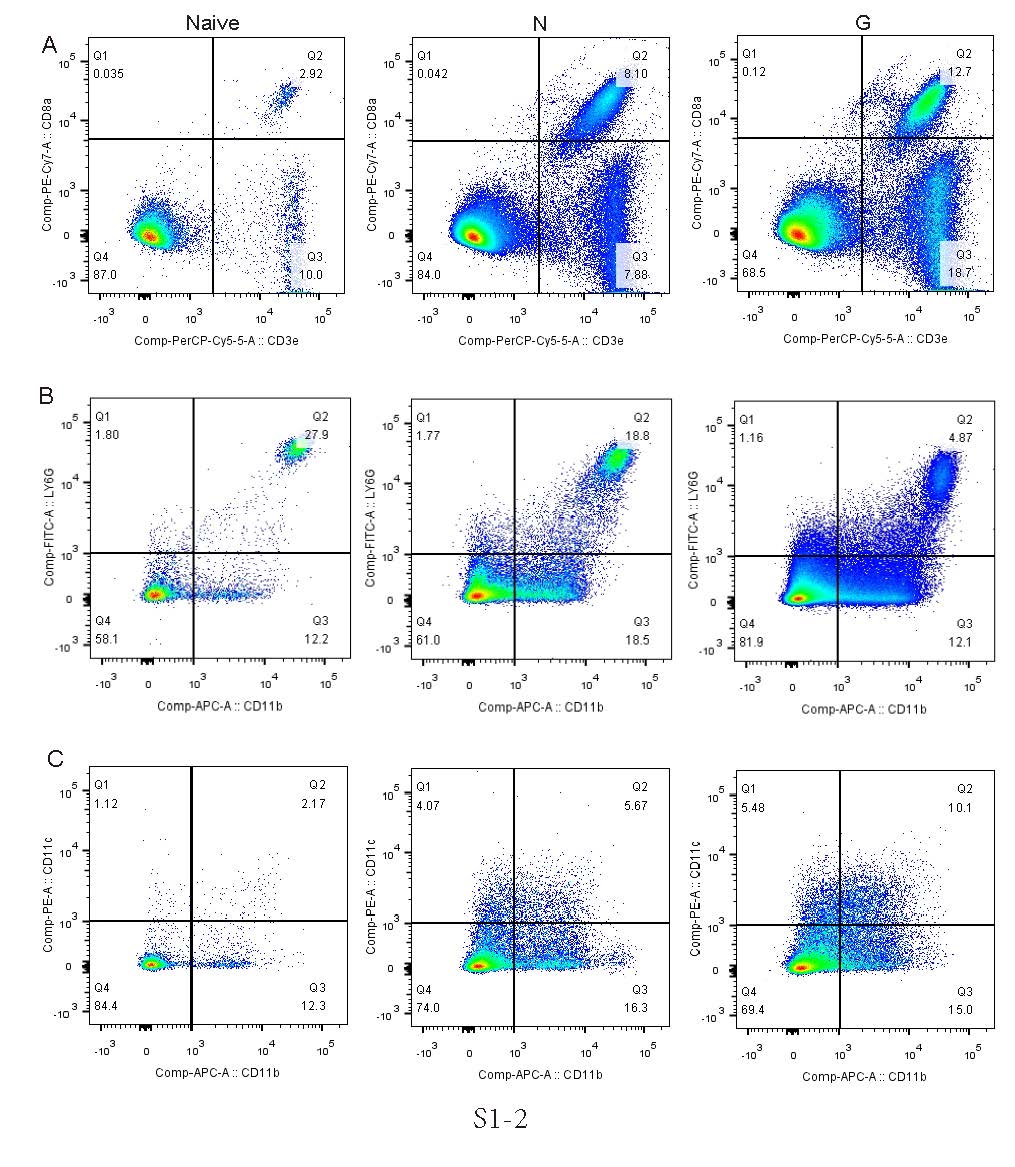

Supplement: Supplementary 1-2 — Flow cytometry analysis of lung cells of mice treated with Gentamicin and then infected with influenza. Gentamicin treated mice and control mice were inoculated with a sublethal dose (103.5TCID50) of influenza PR8. On day 8 after the inoculations, mice were euthanized, (A) CD8+ T cells, (B) CD11b+Ly6G+ cells, and (C) DCs. N, none/PR8, G, Gentamicin/PR8. [file Image_2.jpeg]

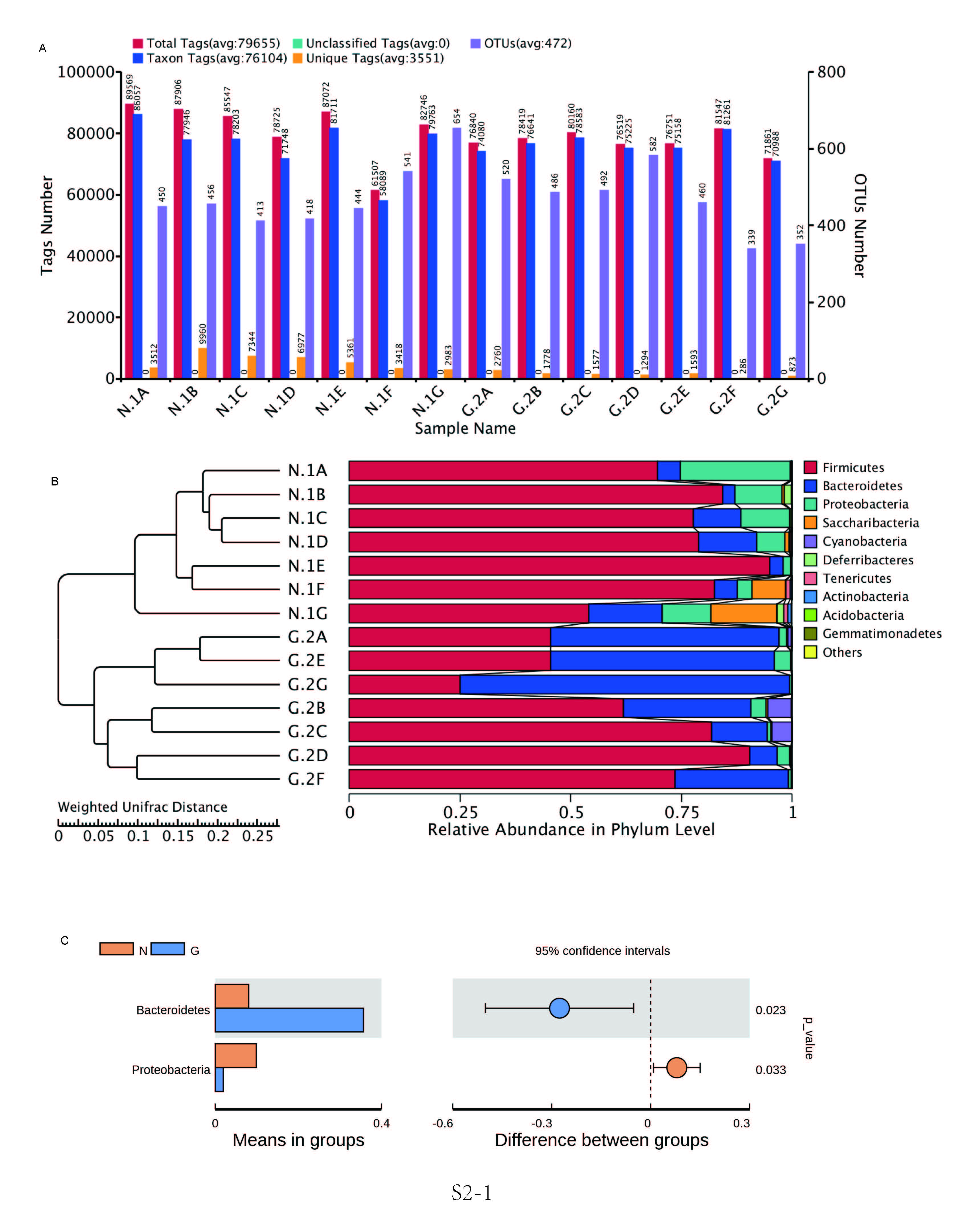

Supplement: Supplementary 2-1 — Gentamicin-induced alteration of colonic microbiota profiles. (A) Tags and outs (B) Relative abundance in phylum levels. (C) Extended error bar plot showing the two bacterial genera with a significant difference (T-test; P< 0.05) in proportions of at least 1% between samples of control group (N) and samples of Gentamicin-treated group (G). One genera(Bacteroidetes)is over abundant within the colonic microbiotas collected from mice treated with Gentamicin compared to those collected from control mice, genera abundance (left) and 95% confidence intervals (right), were detected in 7 Getamicin-treated mice. Number of mice: Control, 7; Gentamicin treated group, 7. N, none/PR8, G, Gentamicin/PR8. [file Image_3.jpeg]
