# Supplementary material for: Gentamicin Induced Microbiome Adaptations Associate With Increased BCAA Levels and Enhance Severity of Influenza Infection
Source: Front Immunol. 2021 Feb 23;11:608895. doi: 10.3389/fimmu.2020.608895 (PMC7940682; doi:10.3389/fimmu.2020.608895)
Supplement: Supplementary 2-2 — Metabolite assignments and chemical shifts of distinguishable peaks. [file Table_1.docx]

**Supplementary 2-2. Metabolite assignments and chemical shifts of distinguishable peaks.**

| No. | Chemical shift (ppm) | VIP/Comp.1 | Metabolite compound | N/G# | Trend |
| --- | --- | --- | --- | --- | --- |
| 1 | 2.3 | 1.7823 | 3-Hydroxybutyrate | 1.7055 | ↑ |
|  | 1.18 | 2.8541 | 3-Hydroxybutyrate | 0.73366 |  |
| 2 | 1.46 | 3.4307 | Alanine | 0.7214 | ↓↓ |
|  | 1.5 | 2.5031 | Alanine | 0.72626 |  |
| 3 | 3.18 | 2.2876 | Choline | 1.1192 | ↑ |
| 4 | 3.02 | 1.6119 | Creatine | 0.91181 | ↓ |
|  | 3.94 | 1.3968 | Creatine | 0.94066 |  |
| 5 | 3.94 | 1.3968 | Glycolate | 0.94066 | ↓ |
| 6 | 1.18 | 2.8541 | Ethanol | 0.73366 | ↓↓ |
| 7 | 3.82 | 1.943 | Glucose | 0.93622 | ↓ |
|  | 3.9 | 1.899 | Glucose | 0.94615 |  |
|  | 3.74 | 1.8458 | Glucose | 0.94386 |  |
|  | 3.7 | 1.5833 | Glucose | 0.94519 |  |
|  | 3.86 | 1.3632 | Glucose | 0.93931 |  |
|  | 3.46 | 1.2961 | Glucose | 0.95922 |  |
|  | 5.22 | 1.0791 | Glucose | 0.95173 |  |
|  | 3.78 | 2.8669 | Glucose | 0.92789 |  |
| 8 | 2.34 | 1.1707 | Glutamate | 1.1859 | ↑↑ |
| 9 | 3.58 | 1.4921 | Glycerol | 1.0749 | ↓ |
|  | 3.66 | 3.534 | Glycerol | 0.85625 |  |
| 10 | 3.58 | 1.4921 | Threonine | 1.0749 | ↑ |
| 11 | 3.54 | 1.4892 | Glycine | 0.95634 | ↓ |
| 12 | 1.34 | 7.1953 | Lactate | 1.1025 | ↑↑ |
|  | 4.1 | 3.4176 | Lactate | 1.0779 |  |
|  | 4.14 | 2.7005 | Lactate | 1.1839 |  |
|  | 1.3 | 2.582 | Lactate | 1.0337 |  |
| 13 | 1.74 | 1.3296 | Arginine | 0.82922 | ↓↓ |
|  | 1.7 | 1.005 | Arginine | 0.88094 |  |
| 14 | 0.94 | 1.3172 | Leucine | 0.92766 | ↓↓ |
|  | 1.74 | 1.3296 | Leucine | 0.82922 |  |
|  | 1.7 | 1.005 | Leucine | 0.88094 |  |
| 15 | 0.94 | 1.3172 | Isoleucine | 0.92766 | ↓ |
|  | 1.02 | 1.1844 | Isoleucine | 0.87081 |  |
| 16 | 3.34 | 1.2216 | Methanol | 1.1454 | ↑↑ |
| 17 | 2.38 | 1.3638 | Pyruvate | 1.174 | ↑↑ |
| 18 | 3.22 | 2.0213 | sn-Glycero-3-phosphocholine | 0.96119 | ↓ |
|  | 3.66 | 3.534 | sn-Glycero-3-phosphocholine | 0.85625 |  |
| 19 | 3.26 | 1.654 | Taurine | 1.0405 | ↑ |
| 20 | 3.26 | 1.654 | Betaine | 1.0405 | ↑ |
| 21 | 0.98 | 1.6536 | Valine | 0.88338 | ↓↓ |
|  | 1.02 | 1.1844 | Valine | 0.87081 |  |

#Average area of N group/average area of G group,>1 instead of levels of N group increased. N, none, G, Gentamicin.
